# Genetic Identification of Medicinal Citrus Cultivar ‘Local Juhong’ Using Molecular Markers and Genomics

**DOI:** 10.3390/genes15060719

**Published:** 2024-06-01

**Authors:** Peng Chen, Jingbo Liu, Qi Tang, Tie Zhou, Lingxia Guo, Yuanyuan Xu, Lijun Chai, Qiang Xu, Ziniu Deng, Xianxin Li

**Affiliations:** 1 Hunan Academy of Agricultural Sciences, Changsha 410125, China; chenpeng@hunaas.cn (P.C.); ccppmuer@hunaas.cn (J.L.); zhoutie0809@163.com (T.Z.); guolingxia@hunaas.cn (L.G.); yuanyuan_xu@hunaas.cn (Y.X.); 2 College of Horticulture, Hunan Agricultural University, Changsha 410128, China; tangqi@hunau.edu.cn; 3 Yuelushan Laboratory, Changsha 410125, China; 4 National Key Laboratory for Germplasm Innovation & Utilization of Horticultural Crops, Huazhong Agricultural University, Wuhan 430070, China; chailijun@mail.hzau.edu.cn (L.C.); xuqiang@mail.hzau.edu.cn (Q.X.)

**Keywords:** medicinal citrus, natural hybrid, molecular markers, genomics, genetic identification

## Abstract

The citrus cultivar ‘Local Juhong’, which has historically been used as a traditional Chinese medicinal material, originated in Yuanjiang County, Hunan Province.Its parental type and genetic background are indistinct as of yet. Morphological observation shows that ‘Local Juhong’ has a slight oblateness in fruit shape, a relatively smooth pericarp, a fine and slightly raised oil vacuole, and an inward concave at the blossom end. The tree form and fruit and leaf morphology of ‘Local Juhong’ are similar to those of ‘Huangpi’ sour orange. To reveal the genetic background of ‘Local Juhong’, 21 citrus accessions were evaluated using nuclear and chloroplast SSR markers and whole-genome SNP information. ‘Local Juhong’ was grouped with mandarins and sub-grouped with ‘Miyagawa Wase’ and ‘Yanxi Wanlu’ in a nuclear SSR analysis, which indicated that its pollen parent might be mandarins. It was closely clustered with orange and pummelo in the chloroplast SSR analysis. The genomic sequence similarity rate of ‘Local Juhong’ with mandarin and pummelo heterozygosity was 70.88%; the main part was the heterozygosity, except for the unknown (19.66%), mandarin (8.73%), and pummelo (3.9%) parts. Thus, ‘Local Juhong’ may be an F_1_ hybrid with pummelo as the female parent and mandarin as the male parent, sharing sisterhood with ‘Huangpi’ sour orange.

## 1. Introduction

Citrus is an economic fruit tree with the largest cultivation area and yield in the world. It has some biological characteristics, such as a long juvenile period, polyembryony, self-incompatibility, and male sterility, that make traditional crossbreeding difficult [1]. Therefore, the development and utilization of natural hybrids or bud mutation varieties have become common breeding methods [2,3]. Yuanjiangcounty is one of the important medicinal citrus production bases in China. ‘Huangpi’ sour orange is the main type and is widely planted in Yuanjiang county and the surrounding area [4]. ‘Local Juhong’ is a local citrus cultivar located in Yuanjiang County, Yiyang City, Hunan Province. The fruitsare oblate with a slightly concaved top, smooth skin, and a dense and slightly convex oil vacuole, and they are mainly used for medicinal and juice processing. Moreover, they have strong tolerance to storage, cold, drought, and disease. However, their genetic background is unknown as of yet.

Hunan Province is one of the most important origincenters of citrus in the world [5,6]. Since the 1950s, many general surveys of citrus resources have been carried out in Hunan Province, and patches of wild oranges in the original secondary forests of Daoxian County, Yizhang County, and Jiangyong County were found [7]. In the 1980s, wild citrus such as Mangshan wild mandarin (*Citrus mangshanensis* S. W. He & G. F. Liu) and Daoxian wild mandarin (*C. daoxianensis* S. W. He & G. F. Liu) were discovered in the Nanling Mountains [8]. After that, they were confirmed as the original types of wild mandarin through extensive research on their classification and evolutionary origin [9,10]. In recent years, the genome of Mangshan wild mandarin was sequenced and assembled, which indicated that the Nanling Mountains may be the center of origin of the wild mandarin germplasm [8]. Hunan Province is also a mixed area for the dissemination of pummelo (*C. maxima*) resources. Numerous local varieties have been found, and their genetic diversity and relationships have also been studied [11,12]. In addition, many other local cultivars and wild resources are also distributed in Hunan, such as sweet orange (*C. sinensis* [L.] Osbeck), sour orange (*C. aurantium* L.), Ichang papeda (*C. ichangensis* Swingle), and Hong Kong kumquat (*Fortunella hindsii* [Champ. Ex Benth.] Swingle) [13,14,15,16].

In the early days, researchers mainly used botany, palynology, and isozyme and karyotype analyses to identify the genetic origin of hybrids, but these methods have some shortcomings, such as large morphological variation, high heterozygosity, and complicated operation [17]. Afterwards, molecular markers were widely used because of their advantages in rapidity, simplicity, high efficiency, and accuracy [18,19,20]. Whole-genome resequencing technology has gradually become a popular method to find variants in sequences, such as single-nucleotide polymorphisms (SNPs), inserts and deletions (Indels), structural variation (SV), and copy number variation (CNV), and analyze individual differences and population genetic characteristics [21,22,23].

We aim to reveal the genetic background and parental relationship of ‘Local Juhong’; the biological characters of ‘Local Juhong’ and ‘Huangpi’ sour orange (*C. aurantium* L.) were measured and analyzed first. Further, twenty accessions from Fortunella, Poncirus trifoliata, and Citrus were chosen for parental identification using nSSR and cpSSR markers. Finally, the genome sequence alignment of ‘Local Juhong’ was contrasted with pummelo, mandarin, navel orange, sour orange, and satsuma mandarin.

## 2. Materials and Methods

### 2.1. Plant Samples

As shown in Table 1, a total of 21 citrus accessions were included. ‘Local Juhong’ was cultivated on Qionghu Street, Yuanjiang County, Yiyang City, Hunan Province, and others were planted in the Hunan Horticultural Research Institute. Fresh leaves were collected and stored at −80 °C for total DNA extraction.

### 2.2. Morphological Traits

A total of 20 morphological traits (tree, leaf, and fruit characteristics) of ‘Local Juhong’ were measured three times in comparison with ‘Huangpi’ sour orange. The data for crown diameter and tree height were obtained by actual measurements of six individual trees. Different characteristics of leaf, fruit, thorn, and spring twig (10 samples each) were measured with a vernier caliper. The average value of each trait was adopted for the analysis. Total soluble solids (TSSs) and titratable acidity (TA) were analyzed with a digital pocket brix-acidity meter (ATAGO, Co., Ltd., Tokyo, Japan). Vitamin C content was determined by titration of 2,6-dichlorophenol indophenol.

### 2.3. DNA Extraction and SSR Primerss

Genomic DNA was extracted from the fresh sample leaves using a modified cetyltrimethylammonium bromide protocol [24]. The DNA concentrations were measured using a Nano Photometer (Shanghai Bio-dl Co., Ltd., Shanghai, China) and diluted to 60 ng/μL. In total, 16 pairs of nSSR primers and 8 pairs of cpSSR primers were used for genetic identification [5,18,25,26]. The primers’ sequence are shown in Table 2 and Table 3.

### 2.4. PCR Amplification and Silver Staining

The PCR reaction system of nSSR and cpSSR was 20 μL in volume, including 10.0 μL × Taq PCR buffer Mix (Shanghai Shenggong, Co., Ltd., Shanghai, China), 0.5 μL forward primer (10 μM), 0.5 μL reverse primer (10 μM), 2.0 μL cDNA (0.5 ng/μL), and 7.0 μL ddH_2_O. The amplification program was as previously described [25]. PCR amplification was conducted in a VeritiTM PCR protocol (Applied Biosystems, Singapore). The amplification effects were verified with 2% agarose gel electrophoresis. The amplicons were separated using denatured 6% polyacrylamide gel electrophoresis and colored with silver stain according to the procedures developed by Biswas et al. [26].

### 2.5. Allele Scoring and Data Analysis

The band statistics and data analysis referred to previous reports. The polymorphic bands of each pair of nSSR and cpSSR primers were counted using the data matrix (0, 1), where 1 stands for having a band, with 0 for no band. The similarity coefficients between samples was calculated by NTSYS-PC 2.1 software; cluster analysis was performed and a phylogenetic tree was constructed by the UPGMA method [27,28].

### 2.6. Genome Resequencing and Analysis

Young leaves of ‘Local Juhong’ were used for 30× depth genome resequencing. Quality control was performed on the raw resequencing data, followed by filtering using the Fast QC software (v0.12.1). After removing connector sequences and low-quality reads, quality-controlled reads were compared with the sweet orange genome using BWA software (version 0.6) [29]. Finally, the sequence alignment of generated data files was carried out via “Citrus ID” to analyze their genome sequence components and genetic background [8].

## 3. Results

### 3.1. Morphological Trait Analysis

**Figure 1:**
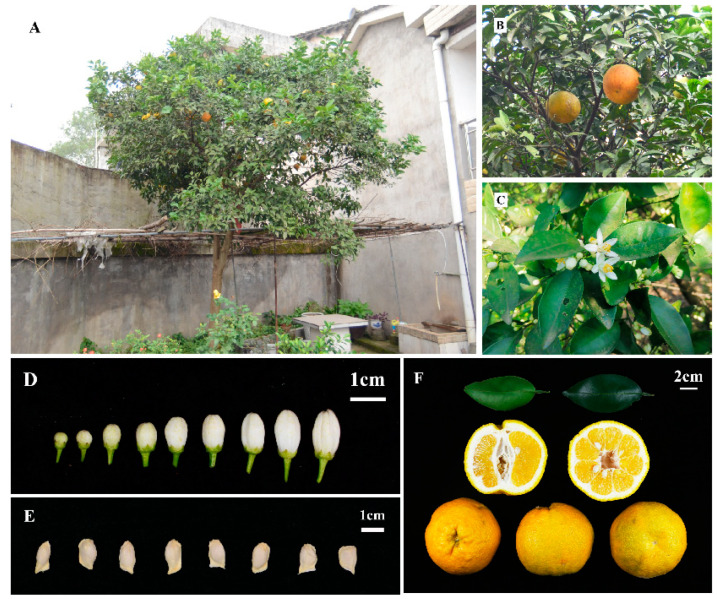
Morphological features of ‘Local Juhong’. (**A**) Tree. (**B**) Fruits on the tree. (**C**) Flowers. (**D**) Flowers at different development stages. (**E**) Seeds. (**F**) Leaves and fruits.

The biological characteristics of ‘Local Juhong’ (J) are shown in Figure 1. As the correlation data in Table 4, the average height of the measured trees of ‘J’ (4.62 ± 0.15 m) was shorter than that of ‘Huangpi’ sour orange (S) (5.03 ± 0.28 m), and the crown diameter of ‘J’ (4.2 × 4.8 m) was also smaller than that of ‘S’ (5.0 × 5.4 m). The length of the spring twigs and thorns of ‘J’ (7.31 ± 1.20 cm, 0.42 ± 0.04 cm) was significantly shorter than that of ‘S’ (19.43 ± 1.55 cm, 1.01 ± 0.31 cm). Few differences were observed in the leaf characteristics of the two cultivars. The leaf length, width, and thickness of ‘J’ are 76.5 ± 4.0 mm, 43.2 ± 2.9 mm, and 0.25 ± 0.01 mm, respectively, while those of ‘S’ are 72.5 ± 6.2 mm, 41.7 ± 2.9 mm, and 0.25 ± 0.02 mm. The length and width of the petiole wing of ‘J’ are 10.8 ± 1.2 mm and 3.6 ± 0.6 mm, and those of ‘S’ are 12.7 ± 1.6 mm and 4.5 ± 1.2 mm (Table 4). The fruit characteristics of the three cultivars were similar, except that the equatorial diameter of ‘J’ (91.0 ± 4.0 mm) is noticeably wider than that of ‘S’ (85.9 ± 2.7 mm). As a result, the fruit shape index of ‘J’ (0.88 ± 0.06) is significantly smaller than that of ‘S’ (0.95 ± 0.07). The fruit surface color and juice color of ‘J’ and ‘S’ are both yellow, but the pericarp surface of ‘S’ is coarser than that of ‘J’. Their fruit maturity period is from November to December. The contents of soluble solids and titratable acid of ‘J’ are 10.0 ± 0 and 1.80 ± 0.01, and those of ‘S’ are 9.4 ± 0 and 1.91 ± 0.02, respectively. The vitamin C content of ‘J’ is 14.8 ± 0.22 mg/mL and that of ‘S’ is 19.0 ± 0.18 mg/mL (Table 4). Generally, regarding the spring twigs, thorns, and petiole wing, those of ‘J’ are significantly shorter than those of ‘S’. The fruit equatorial diameter of ‘J’ is noticeably wider than that of ‘S’, which mainly results in fruit shape difference. Also, there are some little differences in the leaf characteristics, but these are not that obvious.

### 3.2. Phylogenetic Analysis Based on cpSSR and nSSR Markers

**Figure 2:**
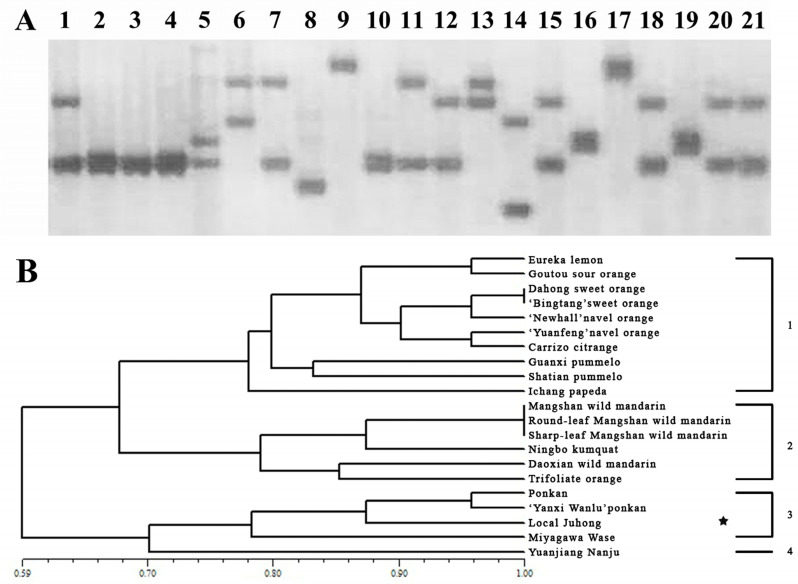
Phylogenetic analysis based on nuclear SSR marker. (**A**) Silver-stained polyacrylamide gel showing polymorphism among 21 tested citrus genotypes and related genera by using the nSSR primer Ma6_64. Materials 1–21 are listed in Table 1. (**B**) UPGMA dendrogram analysis of the 21 citrus genotypes and related genera based on 16 nSSR markers. The asterisk refers to the ‘Local Juhong’ sample.

In total, 16 pairs of nSSR primers were used for the genetic analysis of 21 accessions of citrus and its related genera. The results showed that the 16 pairs of nSSR primers had good polymorphism, and 147 bands were amplified, with an average of 9.19 bands per locus (Table 3). According to the cluster analysis, 21 samples were divided into four groups, and ‘Local Juhong’ was mainly clustered with wide-skinned citrus, clustered with Miyagawa Wase and ‘Yanxi Wanlu’ ponkan in small subgroups, indicating that its male parent may be mandarin (Figure 2).

**Figure 3:**
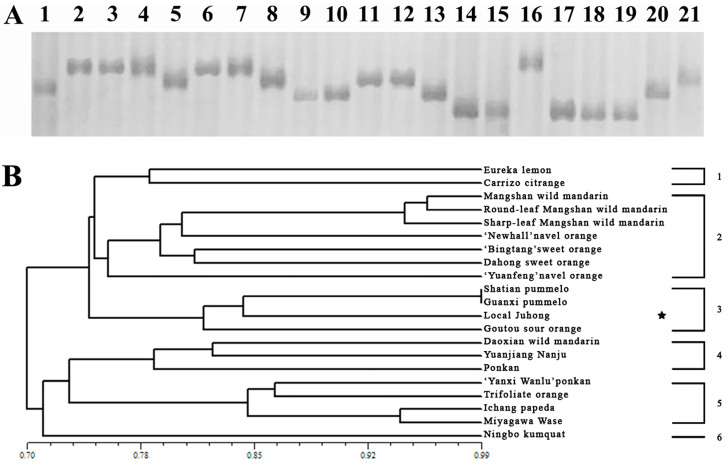
Phylogenetic analysis based on chloroplast SSR marker. (**A**) Silver-stained polyacrylamide gel showing polymorphism among 21 tested citrus genotypes and related genera by using the cpSSR primer ARCP2. Materials 1–21 are listed in Table 1. (**B**) UPGMA dendrogram analysis of the 21 citrus genotypes and related genera based on 8 cpSSR markers. The asterisk refers to the ‘Local Juhong’ sample.

Eight pairs of cpSSR primers were used for the genetic analysis of the 21 samples mentioned above. The results showed that the eight pairs of cpSSR primers had good polymorphism, and a total of 37 bands were amplified, with an average of 6.17 bands per locus. Cluster analysis based on UPGMA showed that 21 samples were divided into six groups, and ‘Local Juhong’ was mainly clustered with oranges and pummelo, indicating that its female parent was closely related to oranges and pummelo (Figure 3).

### 3.3. Genomic Sequence Analysis

**Figure 4:**
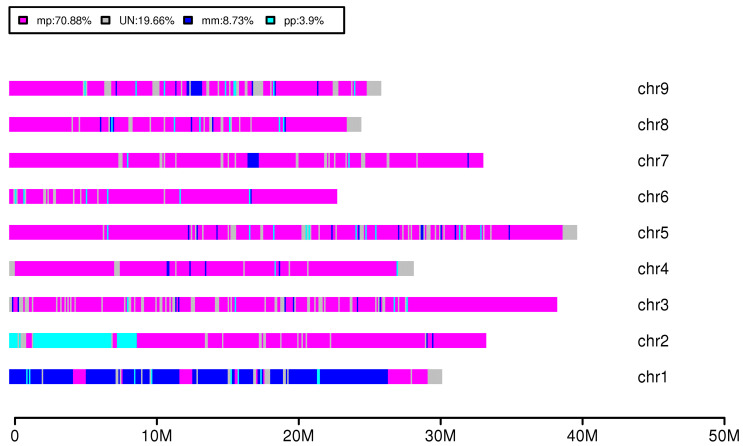
‘Local Juhong’ genome admixture pattern. Blue indicates mandarin background, cyan represents pummelo background, magenta represents hybrid of mandarin and pummelo background, and grey represents unknown part.

**Figure 5:**
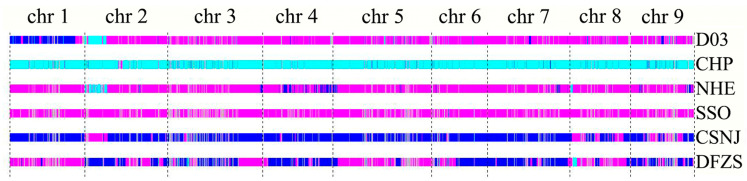
Genome structure of ‘Local Juhong’ and other citrus species. Blue indicates mandarin background, cyan represents pummelo background, magenta represents hybrid of mandarin and pummelo background, and grey represents unknown part. D03 is ‘Local Juhong’, CHP is ‘Chanler’ pummelo, NHE is ‘Newhall’ navel orange, SSO is sour orange from Hunan, CSNJ is a mandarin from Hunan, and DFZS is satsuma mandarin (*C. unshiu*).

Whole-genome analysis of ‘Local Juhong’ indicated that the similarity rate between ‘Local Juhong’ and the mandarin and pummelo heterozygosity was 70.88%, followed by 8.73% similarity to mandarin, 3.9% with pummelo, and 19.66% with an unknown part. Sequence alignment showed that the main gene component of ‘Local Juhong’ is a common component of mandarin–pummelo hybridity (Figure 4). Sequence analysis from nine chromosomes compared with five citrus accessions showed that the genome sequence of ‘Local Juhong’ was highly similar to that of sour orange (SSO); its chromosome 1 was a mainly mandarin sequence, the left side of chromosome 2 was similar to Newhall navel orange and Nanju, and other chromosomes were common components of mandarin–pummelo hybridity (Figure 5). Research has shown that sour oranges are hybrids of mandarin and pummelo (Wang et al., 2021), so we presumed that ‘Local Juhong’ may also be an F_1_ hybrid of mandarin (as the male parent) and pummelo (as the female parent).

## 4. Discussion

The cultivation of sour oranges around Dongting Lake was recorded as early as the Warring States Period [4]. Yuanjiang County is located near Dongting Lake and is rich in citrus resources, such as ‘Zhilugan’, ‘Sainangan’, ‘Shibingju’, ‘Dongtingred’ and ‘Yaojuhong’, among others. Among them, ‘Local Juhong’ may be a variety of ‘Huangpi’ sour oranges, ‘Zhilugan’ and ‘Sainangan’ may be mandarin–orange hybrids, ‘Shibingju’, ‘Dongtingred’ and ‘Yaojuhong’ may be pummelo or sour orange hybrids, and there are more complicated types of hybrids with orange, pummelo, and mandarin, such as ‘Zhenzhiqiao’ and ‘Qiucheng’ [4]. The distribution of local sour oranges and their varieties and hybrids has been recorded in the literature. Our investigation showed that ‘Huangpi’ sour orange was the main cultivar in Yuanjiang County; ‘Local Juhong’ was less distributed, and most of them are old trees. In their production, dried young fruits are made into Chinese herbal medicine (bitter orange), and mature fruits can be used to make juice.

Due to the high specificity, high polymorphism, and stability of SSR molecular markers, many researchers have used them to identify the genetic relationships and genetic diversity of species [30,31,32]. cpSSR is a matrilineal and conservative marker, making it an effective method to identify the source of female parents in citrus [1,25]. In this study, nSSR and cpSSR markers were used, and primers showed good polymorphism. The nSSR marker indicated that ‘Local Juhong’ mainly gathers with mandarin and ponkan and gathers in small subgroups with ‘Miyagawa Wase’ and ‘Ponkan Mandarin’, indicating that its male parent may be mandarin. cpSSR analysis showed that ‘Local Juhong’ mainly clustered with oranges and pummelos, indicating that its female parent was closely related to orange and pummelo. Genome resequencing and sequence alignment showed that the main gene component of ‘Local Juhong’ was a common component of mandarin–pummelo hybridity, and their similarity rate was as high as 70.88%. A previous study showed that sour oranges were an F_1_ hybrid of mandarin and pummelo [33]. So, we speculated that ‘Local Juhong’ may also be an F_1_ hybrid of mandarin (as the male parent) and pummelo (as the female parent), and has a sister relationship with ‘Huangpi’ sour oranges. In industry, sour oranges and their cultivated varieties have been widely considered and applied as Chinese medicinal materials. In this study, systematic scientific research showed that ‘Local Juhong’ is basically a type of sour orange. With its comprehensive tolerance and resistance, ‘Local Juhong’ can definitively be planted and used for medicinal purposes.

Landraces, wild relatives, wild species, genetic stock, advanced breeding material, and modern varieties are some important plant genetic resources [34]. The environment can affect the biological characteristics of plants, leaves, branches, etc. [35]. Plants grow vigorously in environments with sufficient light, good fertilizer, and water. Their branches are thick and short, the leaves are thicker, and their color is lighter. On the contrary, in adverse conditions, plants’ growth potential is weak; their branches are more slender, the leaves are larger and thinner, and the color will be darker. The single-fruit weight would be significantly different because of the different fruit load per plant [36]. Single-fruit weight under many fruits per plant would be smaller and uniform. Fruit quality is also affected by fertilizer and region [37]. Therefore, biological diversity could not be used as effective evidence in genetic identification due to its data being easily affected by the environment. Molecular markers and genome sequences could be used as a scientific and reliable basis for parental identification because of their strong genetic stability. SSR molecular markers have the characteristics of strong specificity, good polymorphism, and co-dominant inheritance. They could be used to identify parent types, evolutionary origins of unknown materials, and genetic diversity [30,31]. cpSSR is matrilineal and conservative, which make it an effective method to identify the source of female parents in citrus [1,25]. Molecular markers and phylogenetic classification could be used to identify parents of unknown materials, but their genomic similarity ratio is still unknown. So, molecular markers could identify basic parent types, but they could not clarify whether backcrossing occurs between offspring and parents. With the maturation of sequencing technology and reduction in costs, genome composition analysis and sequence alignment are widely used. The similarity ratios of sequences were identified using genome-wide SNP and sequence alignment, providing further evidence for parent identification [10,38]. Therefore, they can provide scientific and reliable evidence of the parents and genetic identification of citrus hybrids, facilitating comprehensive analysis of phenotypic data and historical records, molecular markers, and whole-genome sequences.

## 5. Conclusions

In this study, we identified anatural citrus hybrid, ‘Local Juhong’, which waslocated in Yuanjiang County.To reveal its parent types and genetic background, morphological observation, nuclear and chloroplast SSR markers, and genomic sequence alignment analyses were comprehensively applied. The results showed that ‘Local Juhong’ may be an F_1_ hybrid with pummelo as the female parent and mandarin as the male parent, and has a sister relationship with ‘Huangpi’ sour orange.

## Figures and Tables

**Table 1:** List of citrus genotypes and related species used for the analyses.

Code	Common Name	Scientific Name	Source
1	Eureka lemon	*C. limon* (L.) Burm.f.	Horticultural Research Institute of Hunan Province
2	Mangshan wild mandarin	*C. mangshanensis*	Yizhang County, Chenzhou City, Hunan Province
3	Round-leaf Mangshan wild mandarin	*C. mangshanensis*	Horticultural Research Institute of Hunan Province
4	Sharp-leaf Mangshan wild mandarin	*C. mangshanensis*	Horticultural Research Institute of Hunan Province
5	Daoxian wild mandarin	*C. daoxianensis*	Dao County, Yongzhou City, Hunan Province
6	Guanxi pummelo	*C. maxima* (Burn.) Merr.	Horticultural Research Institute of Hunan Province
7	Shatian pummelo	*C. maxima* (Burn.) Merr.	Horticultural Research Institute of Hunan Province
8	Dahong sweet orange	*C. sinensis* (L.) Osbeck	Horticultural Research Institute of Hunan Province
9	Goutou sour orange	*C. aurantium* L.	Horticultural Research Institute of Hunan Province
10	Miyagawa Wase	*C. unshiu* Marc	Horticultural Research Institute of Hunan Province
11	Ponkan	*C. reticulata* Blanco	Horticultural Research Institute of Hunan Province
12	Yuanjiang Nanju	*C. tangerina* Tanaka	Yuanjiang County, Yiyang City, Hunan Province
13	Trifoliate orange	*Poncirus trifoliata* (L.) Raf	Horticultural Research Institute of Hunan Province
14	‘Yuanfeng’ navel orange	*C. sinensis* (L.) Osbeck	Horticultural Research Institute of Hunan Province
15	Carrizo citrange	*C. sinensis* × *P. trifoliata*	Horticultural Research Institute of Hunan Province
16	Ningbo kumquat	*Fortunella crassifolia* Swingle	Horticultural Research Institute of Hunan Province
17	‘Newhall’ navel orange	*C. sinensis* (L.) Osbeck	Horticultural Research Institute of Hunan Province
18	Ichang papeda	*C. ichangensis* Swingle	Horticultural Research Institute of Hunan Province
19	‘Yanxi Wanlu’ ponkan	*C. reticulata* Blanco	Horticultural Research Institute of Hunan Province
20	‘Bingtang’ sweet orange	*C. sinensis* (L.) Osbeck	Horticultural Research Institute of Hunan Province
21	Local Juhong	*C. aurantium* L.	Yuanjiang County, Yiyang City, Hunan Province

**Table 2:** Nuclear SSR markers.

Primer	Motif	Forward Primer 5′–3′	Reverse Primer 5′–3′	Number of Alleles
CS_010	(AAT)_8_	TGCTGCTGCTGCTTCTTCTA	TACCAAGCATTCTGCTGCTG	15
CS_014	(AGA)_8_	GAAGAAGATGGCTGCTCACC	TTCATCATCCTGCCAAGACA	11
CS_018	(TTC)_6_	CCACTCAGCGTTGTTTCAGA	GCGTGTGTGTGTGTGTGTGT	10
CS_024	(TTCT)_6_	TCCTGGGGTACCCTATTGATT	TAACCTTCACCGATCCCTCA	16
CS_052	(ATT)_7_	CGTTCATCTGGGCTTCTTGT	CCTGATGCGCTGAAACAGTA	16
CS_064	(GTT)_7_	TCGATTTCGAGCACTCCTCT	TTCATTCCTCGCGAATAAGC	10
CS_065	(TCT)_6_	CCTCTCTCCCCAGAACTCCT	TTGAGTTTTGATTGAAGCTTTG	8
Csin.0380	(ATA)_10_	GCGAACGAAAGTGAAGGGTA	TTGAACTGCCTGAGTTGTGG	12
Csin.0514	(TGA)_15_	GATGAATCTTTGCCTCTCGC	CTCACAGCCCTTGGTTTGAT	11
Ma2_345	(AT)_6_	AACAATCGGCAACTCCAATC	AGCCATTGAAGGAATGATCC	5
Ma3_80	(AAG)_8_	TGATGGCTTTCGAGTCACTG	CCTATGTAAAGCCTCGCTGC	8
Ma3_143	(TCT)_8_	AATTTGTTGCTGTGCTTCCC	GATCTGGGTTGGATCCTTGA	13
Ma3_177	(GCC)_9_	TCAATTCTATGGTGACCGCA	GCACCGGAAAGTATCCTTCA	10
Ma4_156	(TTTC)_8_	TCGCCTTCTCTCATACACACTG	TGGATGTCTTGCATTCCGTA	4
Ma6_2	(AACGCC)_5_	GGATTTTCGCCACGTGTAAT	GCCGCGAAAATAGACTAACG	11
Ma6_64	(GCACCG)_7_	GACACTTTGGTGGAAATGGG	TTCTGTTGCTGGTTTTGGCT	14

**Table 3:** Chloroplast SSR markers.

Primer	Motif	Forward Primer 5′–3′	Reverse Primer 5′–3′	Number of Alleles
CP1	A_15_	AACGGAAAGAGAGGGATTCG	ACGGGCTTTTTCAAGCATTA	7
CP2	T_12_	TCGTATTCTCGAACCCCTTTT	ATAAATTGCATGGCCGTACC	4
CP4	A_18_	GCTATCCGCCAAGGTAAAGT	TTGAGGTCACGGGTTCAAAT	8
CP6	A_11_	TCAAATGGGTTTGAGGTTGA	GGCGTCCAAAATGCCTATAA	11
CP17	T_10_	TGGTCTAACTCGCCGAATCT	CGGTCAGTAGACCCTGCATT	8
NTCP9	T_10_	CTTCCAAGCTAACGATGC	CTGTCCTATCCATTAGACAATG	3
CCMP2	A_11_	GATCCCGGACGTAATCCTG	ATCGTACCGAGGGTTCGAAT	2
ARCP2	A_13_	TGGAGAAGGTTCTTTTTCAAGC	CGAACCCTCGGTACGATTAA	4

**Table 4:** Fruit morphological traits.

Character	Local Juhong	‘Huangpi’ Sour Orange
Crown diameter (m)	4.2 × 4.8	5.0 × 5.4
Tree height (m)	4.62 ± 0.15	5.0 ± 0.28
Tree age (y)	11	20
Spring twig (cm)	7.31 ± 1.20 a	19.43 ± 1.55 c
Thorn length (mm)	4.2 ± 0.4 a	10.1 ± 3.1 b
Leaf length (mm)	76.5 ± 4.0 a	72.5 ± 6.2 b
Leaf width (mm)	43.2 ± 2.9 a	41.7 ± 2.9 b
Leaf thickness (mm)	0.25 ± 0.01 a	0.25 ± 0.02 a
Length of petiole wing (mm)	10.8 ± 1.2 a	12.7 ± 1.6 b
Width of petiole wing (mm)	3.6 ± 0.6 a	4.5 ± 1.2 b
Fruit maturity	November–December	November–December
Fruit weight (g)	305.8 ± 20.3	273.6 ± 21.5
Fruit polar diameter (mm)	80.1 ± 3.9	81.2 ± 6.7
Fruit equatorial diameter (mm)	91.0 ± 4.0	85.9 ± 2.7
Fruit shape index	0.88 ± 0.06	0.95 ± 0.07
Pericarp thickness (mm)	6.59 ± 1.4 a	7.1 ± 0.6 a
Fruit segment	10.0 ± 0.8	10.0 ± 0.5
Seed number	26.8 ± 3.1	28.8 ± 7.9
Peeling	Easy	Easy
Pericarp surface	Rough	Rough
Fruit surface color	Yellow–orange	Yellow–orange
Juice color	Yellow	Yellow
TSSs (total soluble solids) (%)	10.0 ± 0 a	9.4 ± 0.01 a
TA (titratable acid) (%)	1.80 ± 0.01 a	1.91 ± 0.02 a
Vitamin C (mg·mL^−1^)	14.8 ± 0.22 a	19.0 ± 0.18 b

Note: different lowercases represent difference significance analysis (*p* < 0.05).

## Data Availability

The original contributions presented in the study are included in the article, further inquiries can be directed to the corresponding author.
